# Seroprevalence of SARS-CoV-2 among children visiting a hospital during the initial Seattle outbreak

**DOI:** 10.1101/2020.05.26.20114124

**Published:** 2020-06-02

**Authors:** Adam S Dingens, Katharine HD Crawford, Amanda Adler, Sarah L Steele, Kirsten Lacombe, Rachel Eguia, Fatima Amanat, Alexandra C Walls, Caitlin R Wolf, Michael Murphy, Deleah Pettie, Lauren Carter, Xuan Qin, Neil P King, David Veesler, Florian Krammer, Helen Y Chu, Janet A Englund, Jesse D Bloom

## Abstract

Children are strikingly underrepresented in COVID-19 case counts. In the United States, children represent 22% of the population but only 1.7% of confirmed SARS-CoV-2 cases. One possibility is that symptom-based viral testing is less likely to identify infected children, since they often experience milder disease than adults. To better assess the frequency of pediatric SARS-CoV-2 infection, we serologically screened 1,775 residual samples from Seattle Children's Hospital collected from 1,076 children seeking medical care during March and April of 2020. Only one child was seropositive in March, but nine were seropositive in April for a period seroprevalence of >1%. Most seropositive children (8/10) were not suspected of having had COVID-19. The sera of most seropositive children had neutralizing activity, including one that neutralized at a dilution >1:18,000. Therefore, among children seeking medical care, the frequency of SARS-CoV-2 infection increased markedly during the early Seattle outbreak despite few positive viral tests.

One of the first cases of community transmission of SARS-CoV-2 in the United States was identified in the greater Seattle area in late February, 2020[8,9]. By late March, thousands of cases had been identified in Washington state by viral RT-PCR testing, mostly among adults (https://www.doh.wa.gov/Emergencies/Coronavirus). Schools closed statewide on March 17, and a statewide stay-at-home order was issued the next week. March and April of 2020 are therefore critical months for understanding the early dynamics of the SARS-CoV-2 pandemic in the Seattle area.

Because SARS-CoV-2-infected children often experience little or no disease[1,4–6], we sought to identify infections using an approach independent of symptom-based viral testing.

Serological assays, which detect antibodies induced by infection, provide such an approach. When interpreting these assays in a temporal context, note that individuals do not become seropositive until ≈1 to 2 weeks post symptom onset[10–14].

We serologically screened 1,775 residual serum samples from Seattle Children's Hospital that were collected between March 3 and April 24, 2020 following approval from the Human Subjects Institutional Review Board. These samples were collected from 1,076 unique children who visited the hospital and received blood draws for any reason, including respiratory illnesses, surgery, or ongoing medical care. Demographics and reason for medical admission are presented below with results of our serological testing. The generalizability of this study population to all children in Seattle is unknown, particularly because hospital visitors were primarily those with urgent medical needs during the statewide stay-at-home order.

We used a multi-assay serological testing approach based on an enzyme-linked immunosorbent assay (ELISA) protocol that recently received emergency use authorization from New York State and the FDA[15,16], although we added an additional validation assay to increase stringency. Furthermore, as described below, we confirmed that most seropositive samples had activity in pseudovirus neutralization assays.

We first screened all sera at a 1:50 dilution for IgG binding to the SARS-CoV-2 spike receptor binding domain (RBD) and compared results to a negative control consisting of a pool of sera collected in 2017 and 2018 (Figure 1a). We identified 102 of 1,775 samples with readings that exceeded the average of the negative controls by >5 standard deviations. These preliminary hits were further assessed with IgG ELISAs using serial dilutions of sera against two antigens: RBD and pre-fusion stabilized spike ectodomain trimer (Figure 1b). As negative controls, we included twelve serum samples and two serum pools collected before 2020; we also tested some pediatric samples that were negative in the initial RBD screen. We summarized the ELISAs by calculating the area under the curve (AUC), and called samples as seropositive if the AUC exceeded the average of the negative controls by >5 standard deviations for both RBD and spike (Figure 1c). The AUCs for RBD and spike ELISAs were highly correlated (Figure 1d), but some sera exhibited slightly higher reactivity to one antigen than the other, meaning that screening on both antigens introduces additional stringency.

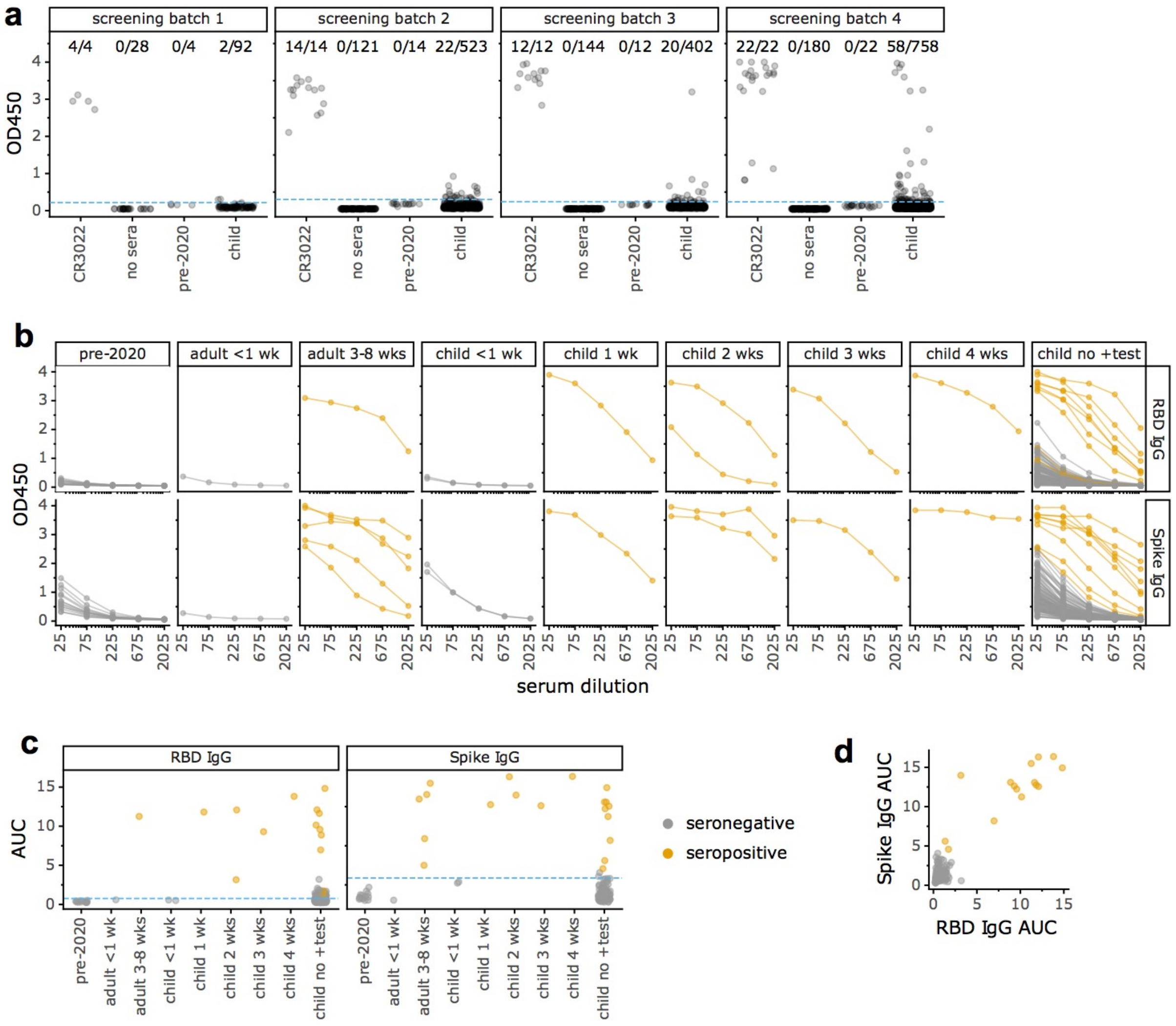

**Figure 1:** Multi-step serological testing. **(a)** We screened 1,775 child samples by ELISA to RBD at a single dilution in four batches, with CR3022 antibody[17,18] as a positive control and pre-2020 sera as a negative control. Samples with OD450 readings that exceeded pre-2020 sera by >5 standard deviations (dotted blue line) were considered potential hits. **(b)** All potential hits from the initial screen as well as some screen-negative samples and additional controls were tested at serial dilutions for binding to RBD (top) or full spike (bottom). Samples from adults or children with RT-PCR-confirmed infections are labeled by weeks post symptom onset; all remaining samples from children with no positive RT-PCR test are in the rightmost facet. Samples were classified as seropositive (orange) if the AUC exceeded pre-2020 negative controls by >5 standard deviations in both assays. **(c)** AUC calculated from panel (b), with dotted blue line indicating the cutoff for that assay. **(d)** Correlation between AUC for RBD and spike (Pearson's r = 0.93). For adult and pre-2020 sera, each curve in (b) or point in (c) represents a unique individual or sera pool. For child samples labeled by week, each facet is a unique individual but "child <1 wk" and "child 2 wks" have two samples collected on the same day; for "child no +test" many unique individuals are shown in the facet. See Methods for more details.

Visual inspection of the ELISA results in Figure 1b,c provides a sense of the tradeoffs in calling seropositivity. Our assays included controls from adults with RT-PCR-confirmed infections, as well as samples from five children with confirmed infections collected at various times post symptom onset. Neither adult nor child samples <1 week post symptom onset were seropositive by our criteria, consistent with prior reports on kinetics of the antibody response to SARS-CoV-2[10–14]. However, all samples ≥1 week post symptom onset were seropositive, and in most cases the signal greatly exceeded pre-2020 negative controls. Samples from children who never tested positive for SARS-CoV-2 are shown at far right in Figure 1b,c. The samples in this set that we classified as seropositive had readings comparable to confirmed infections ≥1 week post symptom onset. However, some samples from children who had not tested positive for virus had readings that exceeded pre-2020 negative controls but were weaker than confirmed-infection samples ≥1 week post symptom onset. Our stringent criteria classify these samples as seronegative, although it is possible that some represent recent infections in children who had not yet developed robust antibody responses. Indeed, one symptomatic child who tested positive by RT-PCR had multiple samples taken <1 week post symptom onset that were seronegative (Figure 1b, "child < 1 wk"). However, in light of concerns about the poor specificity of some serological assays[19–21], we chose to conservatively call only strongly seropositive samples at the potential cost of missing some recent infections.

Overall, our assays identified 12 seropositive samples from 10 different children in the study population. These include 9 seropositive samples from 8 children who had never tested positive for SARS-CoV-2 (far right facet of Figure 1b,c), as well as the samples from the children labeled as 2 and 3 weeks post symptom onset in Figure 1b,c. The seropositive samples from the children 1 and 4 weeks post symptom onset are from RT-PCR-confirmed SARS-CoV-2 infections that were referred to Seattle Children's Hospital, and are not part of the residual serum pool that makes up our study population (see Methods for details).

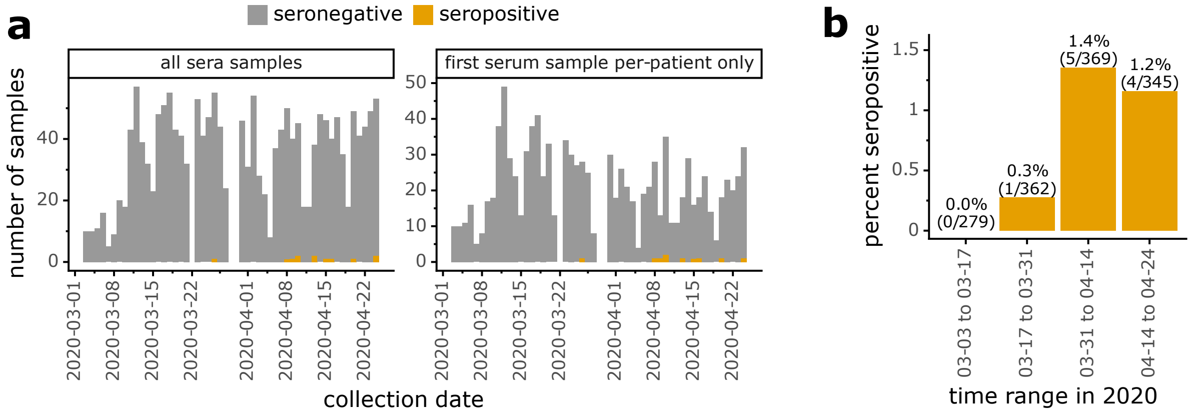

**Figure 2:** Seroprevalence over time. **(a)** Total and seropositive samples collected each day in the study period, with stacked bars showing seropositive samples in orange and seronegative ones in gray. The left panel shows all 1,775 residual samples, while the right panel shows only the first sample from each of the 1,075 patients. **(b)** Period seroprevalence in two-week intervals. Bars show percentage of tested patients with at least one seropositive sample during each period. Seroprevalence was significantly higher from March 31 to April 24 than March 3 to March 31 ($P = 0.02$, Fisher exact test, two-sided).

We next examined the frequency of seropositive samples in the context of the temporal dynamics of the SARS-CoV-2 outbreak in Seattle (Figure 2a). The first seropositive sample was collected in late March, and there were no additional seropositive samples until the second week of April. From that time on, a low but steady fraction of samples were seropositive for a period seroprevalence slightly greater than 1% in April (Figure 2a,b). Notably, our period seroprevalence measurements for children seeking medical care are similar to all-age cumulative incidence estimates for the Seattle region based on viral testing and mortality data[22,23] given the ≈1 to 2 week lag between symptom onset and seroconversion[10–14].

| | | All Children (n=1076) | Seropositive Children (n=10) |
|---|---|---|---|
| Age (years) | 0 to 4 | 192 | 4 |
| | 5 to 9 | 214 | 1 |
| | 10 to 14 | 301 | 2 |
| | ≥15 | 369 | 3 |
| Sex | F | 535 | 4 |
| | M | 541 | 6 |
| RT-PCR viral testing status | Positive | 3 | 2 |
| | Negative | 389 | 3 |
| | Not tested | 684 | 5 |
| Admit type | Outpatient | 653 | 4 |
| | Inpatient | 306 | 4 |
| | Emergency | 101 | 2 |
| | Day surgery | 16 | 0 |

**Table 1:** Cohort demographics. If a child had multiple samples, age and admit were determined based on the child's first visit. For viral testing status, a child was classified as positive if they had a positive viral test at any visit.

We also examined how many seropositive children had received RT-PCR viral tests for COVID-19. This is an important question, since children are under-represented in viral-testing case counts[1–3] and household-contact studies differ regarding whether secondary attack rates are lower in children than adults[24,25]. Over a third of children in our study had received at least one viral test (Table 1; note that administration of a viral test does not imply a child was suspected of having COVID-19, since tests were routinely administered before hospital admission or procedures such as surgery). Of the 10 seropositive children, only 2 had tested positive for virus (Table 1, Supplementary Table 1). Three other seropositive children had tested negative for virus in routine screening prior to surgery. The remaining seropositive children never received a viral test. Additionally, 1 child with a positive viral test was seronegative; this is the child whose serum was collected <1 week post symptom onset (Figure 2b,c), prior to when seroconversion is expected to occur.

A detailed chart review revealed that only the two seropositive children with a RT-PCR confirmed viral infection had documented COVID-19 symptoms (Supplementary Table 1). One additional seropositive child, who presented at the hospital for an allergic reaction, had previous household exposure to the virus but had not been tested because she did not develop symptoms. The seven other seropositive children were at the hospital for reasons unrelated to respiratory illness and had no documented exposures (Supplementary Table 1).

Other demographic and clinical data had no noteworthy associations with seropositivity. Seropositive children were about equally likely to be male or female (Table 1), agreeing with studies finding no major sex differences in pediatric cases in China[4,6], but contrasting with a report of stronger male sex skewing in pediatric oncology patients in New York[26]. The seropositive children spanned all ages from 0-4 years to ≥15 years and were admitted to the hospital for a variety of reasons (Table 1, Supplementary Table 1).

Finally, we measured the neutralizing activity of sera from seropositive children using lentiviral particles pseudotyped with spike[27]. Eight of 10 seropositive children neutralized virus at a >1:25 dilution (Figure 3, Supplementary Figure 1), with neutralization correlating with RBD and spike binding as measured in the ELISAs (Supplementary Figure 2). This frequency of neutralizing activity is consistent with reports that 80-90% of recovered adults have neutralizing activity[10,28,29], as well as our measurements for 7 adults at 3 to 8 weeks post symptom onset (Figure 3). Note that the two children without neutralizing activity had not experienced symptoms, so it is possible that the sera was collected before neutralizing activity is expected to appear[10]. Two children had very high neutralizing activity, including one with a potency that exceeded the limit of our assay (>1:18,000 dilution). In comparison, the aggregated results of four studies of adults reported only 2 out of 263 individuals who had neutralizing activity >1:10,000 at 2 to 5 weeks post symptom onset using lentiviral pseudotype assays similar to our own[10,28–30]. Our sample size is too small to draw conclusions about neutralizing immunity in children versus adults, but this is an interesting area for further study given that children develop stronger or quicker neutralizing responses to some other viruses[31–35].

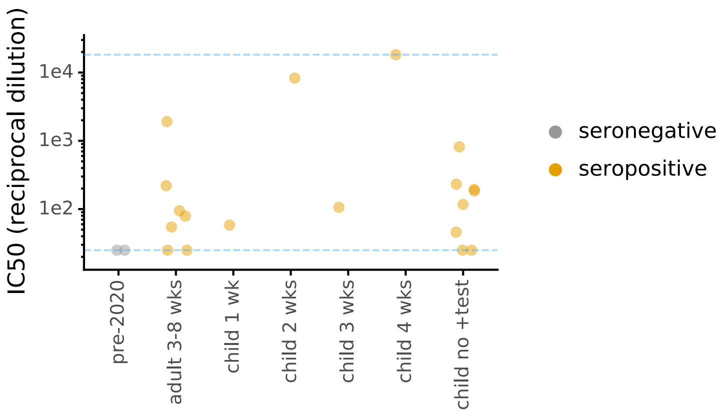

**Figure 3:** Neutralizing activity of sera against Spike-pseudotyped lentiviral particles. The y-axis is the reciprocal dilution of serum that inhibits infection by 50% (IC50). Dashed blue lines are the limits of the dilution series; points at those limits represent lower or upper bounds on the IC50. The child sera shown here are from the same individuals as in Figure 1b,c. Full curves are in Supplementary Figure 1.

In this study, we used serological assays to retrospectively identify SARS-CoV-2 infections in children early in the Seattle outbreak. Although our study used sera collected from children seeking medical care and therefore does not represent an unbiased population survey, it nonetheless represents the first large-scale SARS-CoV-2 serological survey of children. In particular, seropositivity increased markedly from March to April of 2020, and most seropositive children had never tested positive for virus. However, the overall frequency of seropositivity was low (≈1%) even in April, suggesting that while infections of children are often missed by viral testing perhaps due to the lack of symptoms, only a small fraction of children in Seattle had been infected by SARS-CoV-2 as of April 2020.

## *Methods*

*Study participants*

Residual sera samples at Seattle Children's Hospital were collected starting March 3, 2020. Adequate volume of sera remaining after other lab tests were conducted was the main sample-selection criterion, which inherently reduces the relative number of samples from infants who have smaller blood-draw volumes. Adult samples (Figure 1b,c) were residual plasma collected from RT-PCR confirmed infections from the Seattle area at the University of Washington, or from COVID-19-positive individuals enrolled in a prospective cohort study. The sample collection and this study were approved by the Institutional Review Boards of Seattle Children's Hospital and the University of Washington.

Additional RT-PCR-confirmed COVID-19 pediatric cases were actively recruited to enroll in another approved study at Seattle Children's Hospital during this study period. These actively enrolled children were omitted from this seroprevalence study, which consisted of the residual serum sample pool at Seattle Children's Hospital and its clinics. However, pediatric samples from 1 and 4 week(s) post symptom onset facets in Figure 1b,c and Figure 3 are from these actively enrolled children and were included in these plots to illustrate the sensitivity of our serological assays—however, these two children are not included in estimates of seroprevalence Figure 2 or Table 1. Additionally, in Figure 1b,c, samples in the <1 week facet (two samples from same individual), the 2 week facet (two samples from another individual), and the 3 week facet (single sample another individual) were RT-PCR-confirmed cases that were not recruited to Seattle Children's Hospital and so are included in the seroprevalence estimates.

*Serological assays*

We initially screened all sera at a 1:50 serum dilution for IgG binding to RBD. All sera were heat-inactivated at 56°C for 1 hour. Ninety-six well Immunlon 2HB plates (Thermo Fisher; 3455) were coated with 2 μg/mL of His-tagged RBD in phosphate buffered saline (PBS) overnight at 4°C. The RBD antigen was produced in mammalian cells and purified as previously described[15,16,36]. The next day, plates were washed 3 times with PBS containing 0.1% Tween 20 (PBS-T) using an automated plate washer (Tecan HydroFlex) and blocked for 1-2 hours at room temperature with PBS-T containing 3% non-fat dry milk. Sera were diluted 1:50 in PBS-T containing 1% non-fat dry milk. Block was thrown off, and 100 μL of diluted sera was transferred to the ELISA plate in a setup as previously described[16]. Each plate also contained two positive control wells (CR3022[17,18], an anti-SARS-CoV-1 monoclonal antibody that reacts to the SARS-CoV-2 RBD, at 0.5 μg/mL) and two negative control wells (pooled human sera taken from 2017-2018 (Gemini Biosciences, 100-110, lot H86W03J). CR3022 was expressed in Expi293F cells and purified by protein A and size exclusion chromatography using established methods. After a two-hour incubation at room temperature, plates were washed with PBS-T thrice. Goat anti-human IgG-Fc horseradish peroxidase (HRP)-conjugated antibody (Bethyl Labs, A80-104P) was diluted 1:3,000 in PBS-T containing 1% milk and 50 μL was added to each well. After 1 hour at room temperature, plates were washed thrice with PBS-T, and 100 μL of TMB/E HRP substrate (Millipore Sigma; ES001) was added to each well. After 5 minutes, 100 μL 1N HCl was added, and OD450 was read immediately on a Tecan infinite M1000Pro plate reader. Samples were considered potential positive hits in the screen if their reading exceeded the average of all of the 2017-2018 negative control readings by >5 standard deviations, computing this threshold separately for each screening batch (Figure 1a).

Follow-up ELISAs were performed on all potential positive hits from the screening assay, plus a subset of samples that were negative in the initial screen (all samples that proved seropositive by our criteria were positive in the initial screen). These follow-up ELISAs were performed as for the screening step described above, with the following differences: All sera were run at five 3-fold dilutions, starting at 1:25. Each plate contained a negative control dilution series (pooled human sera taken from 2017-2018), and a CR3022 positive control dilution series starting at a concentration of 1 ug/mL. Trimeric, prefusion-stabilized spike was produced as previously described[36] with the following minor changes: the protein was produced using expiHEK293F cells transfected transiently with PEI, and the cultures were grown at 33°C for 3 days prior to downstream talon batch purification. The additional control samples tested during follow up ELISAs were pooled human sera from 2008-2015 (Gemini Biosciences, 100-110, lot H87W00K), and 12 de-identified banked serum samples collected between 1986 and 1992 (Bloodworks Northwest).

*AUC analysis*

For the follow-up ELISAs performed at serial dilutions, the AUC represents the area under the titration curve after putting the serial dilutions on a log-scale (as plotted in Figure 1b). Readings for the 2017-2018 pooled sera that was run on each plate were first averaged and treated as a single sample. This, along with the 12 banked pre-2020 sera samples and the additional 2008-2015 pooled sera were treated as 14 negative controls and used to determine a cutoff in each assay (average of all negative controls plus five standard deviations).

*Neutralization assays*

SARS-CoV-2 spike-pseudotyped lentivirus neutralization assays were performed as previously described[27], with the following slight modifications. Infections were carried out in poly-L-lysine (P4707, Millipore Sigma, Burlington, MA, USA) coated black-walled, clear-bottom plates (655090, Greiner Bio-One, Kremsmünster, Austria) and luciferase activity was measured in these plates without transferring to opaque bottom plates. Sera were diluted 3-fold seven times starting at a 1:25 dilution and luciferase activity was measured at 52 hours post-infection. Target cells were HEK-293T cells transduced to express hACE2[27] (BEI Resources, NR-52511). Samples were run in duplicate and each plate included two no-serum controls. Fraction infectivity was calculated by normalizing the luciferase reading for each sample by the average of the two no-serum control wells in the same row. Neutralization curves were plotted using the `neutcurve` Python package (https://jbloomlab.github.io/neutcurve/, 0.3.1). This package fits a three-parameter Hill curve, with the top baseline fixed to one and the bottom baseline fixed to zero. The neutralization curves are shown in Supplementary Figure 1.

*Period seroprevalence analysis*

Period seroprevalence was calculated over two-week periods at the individual level. We calculated the percentage of all tested patients that had a seropositive sample during each time period. Individuals were counted a single time even if multiple samples from a single time period were tested. If an individual contributed samples to multiple time periods, they were counted for each time period.

*Data availability*

Full raw data for all serological assays, as well as much demographic and viral testing data that can be provided without compromising sample and patient de-identification, is available in Supplementary Data files 1 to 4.

***Acknowledgements***

We are grateful to all study participants for contributing samples. We also thank Abigail Powell, Peter Rupert, and Roland Strong for sharing antigens; Elizabeth Ahearn for laboratory management at

Seattle Children's Hospital; and Mike Famulare and Alex Greninger for helpful comments on this manuscript. This work was supported by the NIAID / NIH (R01AI141707 and R01AI140891 to J.D.B., HHSN272201700059C to D.V., and F30AI149928 to K.H.D.C.), the NIMGS/NIH (R01GM120553 to D.V.), the Bill & Melinda Gates Foundation (OPP1156262 to N.P.K.), a Pew Biomedical Scholars Award (to D.V.) and Burroughs Wellcome Investigators in the Pathogenesis of Infectious Diseases awards (to D.V. and J.D.B.). J.D.B. is an Investigator of the Howard Hughes Medical Institute. Work in the Krammer laboratory was partially supported by the NIAID Centers of Excellence for Influenza Research and Surveillance (CEIRS) contract HHSN272201400008C, Collaborative Influenza Vaccine Innovation Centers (CIVIC) contract 75N93019C00051 and philanthropic donations.

*Author Contributions*

Conceptualization: A.S.D, J.A.E., and J.D.B.; investigation: A.S.D, K.H.D.C., R.E.; analysis: A.S.D., K.H.D.C., and J.D.B.; clinical management, sample and data handling: A.A., S.S., K.L., C.R.W., X.Q., H.Y.C., and J.A.E.; resources and specialized reagents: A.C.W., C.R.W., F.A., N.P.K., D.V., F.K., H.Y.C.; writing—original draft preparation, A.S.D and J.D.B.; writing—review and editing: all authors. All authors have read and agreed to the published version of the manuscript.

*Competing interests*

H.Y.C. is a consultant for Merck and Glaxo Smith Kline and receives research funding from Sanofi Pasteur, outside of the submitted work. N.P.K. is a co-founder, shareholder, and chair of the scientific advisory board of Icosavax, Inc. Mount Sinai has licensed serological assays to commercial entities and has filed for patent protection for serological assays. J.A.E. is a consultant for Sanofi Pasteur and Meissa Vaccines. The other authors declare no conflicts of interest.
