# Supplementary material for: Serological identification of SARS-CoV-2 infections among children visiting a hospital during the initial Seattle outbreak
Source: medRxiv. 2020 Jun 30:2020.05.26.20114124. Preprint. [Version 3] doi: 10.1101/2020.05.26.20114124 (PMC7273251; doi:10.1101/2020.05.26.20114124)
Supplement: Supplement 2020 [file 83214-2020.05.26.20114124-1.pdf]

Supplementary information

Patient ID	Age (years)	Sex	COVID-19 symptoms	Weeks post-symptom onset of first seropositive sample	COVID-19 disease suspected by clinicians	Reported COVID-19 exposure	Mid-nasal specimen tested for SARS-CoV-2 by RT-PCR (result)	Admission type	Clinical notes	RBD IgG ELISA AUC	Spike IgG ELISA AUC	Neutralization IC50 (reciprical serum diltuion)
**11c2f253**	0 to 4	Female	No	N/A	No	Household exposure	No	Emergency	Allergy related visit; previous household exposure but not tested because asymptomatic	6.971	8.185	816.7
**16daf987**	≥15	Male	Yes, previously	3	Known to be previously infected	Household exposure	Yes (positive)	Outpatient	Previously SARS-CoV-2 positive after household exposure. Seen for chronic medical condition unrelated to COVID-19 several weeks later	9.298	12.62	106.2
**614c18c2**	≥15	Female	No	N/A	No	None documented	Yes (negative)	Inpatient	Screened by RT-PCR before surgery	1.729	4.574	<25
**820dcc2b**	0 to 4	Male	No	N/A	No	None documented	No	Emergency	Melena without respiratory symptoms	14.83	14.93	193.9
**90735c96**	≥15	Female	Yes	2	Yes	Household exposure	Yes (positive)	Inpatient	Previously SARS-CoV-2 positive after household exposure. Presented with worsening respiratory symptoms and had an underlying autoimmune disease	12.08	16.32	8277
**a09b7cad**	10 to 14	Female	No	N/A	No	None documented	No	Outpatient	Routine visit for underlying rheumatologic condition	1.346	5.603	<25
**be4c4842**	0 to 4	Male	No	N/A	No	None documented	Yes (negative)	Inpatient	Screened by RT-PCR before surgery for drainage of infection	12.09	12.56	231.9
**d98c7284**	10 to 14	Male	No	N/A	No	None documented	No	Outpatient	Routine visit for underlying cardiac condition	8.874	13.09	116.8
**da9ab30a**	0 to 4	Male	No	N/A	No	None documented	Yes (negative)	Inpatient	Screened by RT-PCR before surgery for drainage of infection	11.63	13.07	184.7
**e014251d**	5 to 9	Male	No	N/A	No	None documented	No	Outpatient	Routine blood draw for underlying cardiovascular condition	10.14	11.24	45.96

Supplementary Table 1: Clinical characteristics of seropositive patients. If a patient had multiple samples (n=2 seropositive patients), age and admission type were determined based on sample from patient's first visit; for serological assays, data from the patient's last sample is shown. A patient was reported as positive for RT-PCR testing if they had ever received a positive result, including at timepoints prior to samples tested in this study.

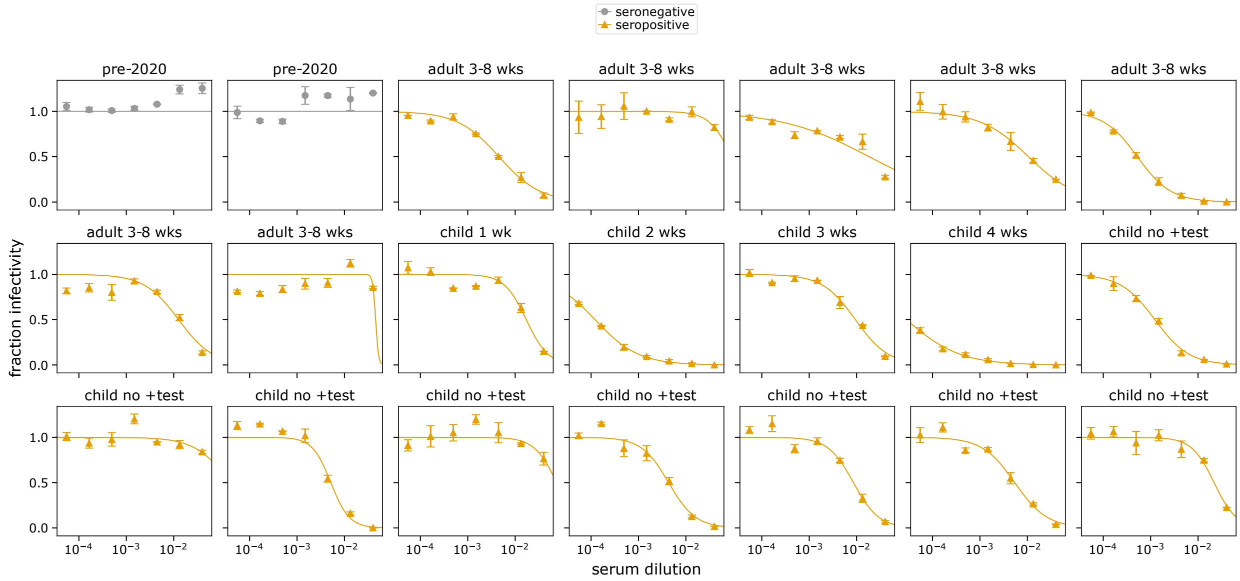

Supplementary Figure 1: Full neutralization curves for all sera that were used to calculate the IC50s plotted in Figure 3. The raw values plotted here are in Supplementary Data 3, and numerical values for the IC50s are in Supplementary Data 4.

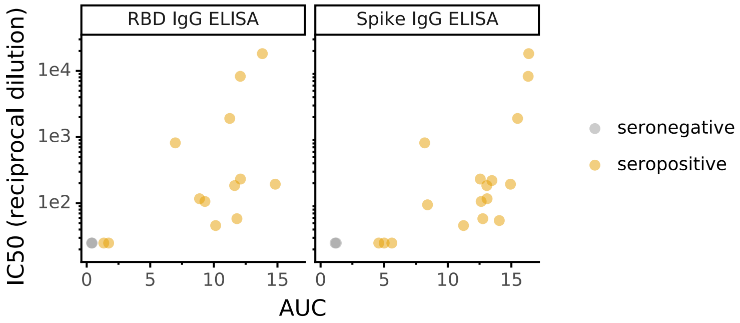

Supplementary Figure 2: Correlation of the IC50s from the neutralization assays to the AUCs measured in the ELISAs for all samples tested in both assays. The numerical values are in Supplementary Data 4.

Supplementary Data 1: De-identified clinical data, raw OD450 values from RBD IgG ELISA screen, and AUCs from follow up ELISAs for each sample.

Supplementary Data 2: Raw OD450 values across all titrations from RBD and Spike follow-up IgG ELISAs.

Supplementary Data 3: Fraction infectivity values across all titrations from Spike-pseudotyped lentivirus neutralization assays.

Supplementary Data 4: Summary IC50 values and RBD and Spike IgG ELISA AUC values for all samples run in Spike-pseudotyped lentivirus neutralization assays.
